# Structure Characterization and Functional Properties of Flaxseed Protein–Chlorogenic Acid Complex

**DOI:** 10.3390/foods12244449

**Published:** 2023-12-12

**Authors:** Weiwei Cao, Junliang Chen, Shuhua Ma, Xin Chen, Xin Dai, Li Zhang, Mengyao Guo, Linlin Li, Wenchao Liu, Guangyue Ren, Xu Duan, Qinggang Xie

**Affiliations:** 1 College of Food and Bioengineering, Henan University of Science and Technology, Luoyang 471023, China; caoweiwei@haust.edu.cn (W.C.); 18848967767@163.com (M.G.);; 2 Heilongjiang Feihe Dairy Co., Ltd., Beijing 100015, China

**Keywords:** FP–phenolic acid complex, functional food, encapsulation via freeze-drying, structure characterization, function

## Abstract

This study aimed to investigate the effects of the covalent binding of flaxseed protein (FP) and chlorogenic acid (CA) on the structure and functional properties of FP–CA complexes fabricated using the alkali method. The results suggested that the encapsulation efficiency of CA encapsulated by FP ranged from 66.11% to 72.20% and the loading capacity of CA increased with an increasing addition ratio of CA with a dose-dependent relationship, which increased from 2.34% to 10.19%. The particle size, turbidity, zeta potential and PDI of FP and the FP–CA complexes had no significant discrepancy. UV–Vis and fluorescence spectra showed the existence of the interaction between FP and CA. SEM images showed that the surface of the FP–0.35%CA complex had more wrinkles compared to FP. Differential scanning calorimetry analysis indicated the decomposition temperature of FP at 198 °C was higher than that (197 °C) of the FP–0.35%CA complex, implying that the stability of the FP–CA complexes was lower than FP. The functional properties suggested that the FP–CA complex with 1.40% CA had a higher water holding capacity (500.81%), lower oil holding capacity (273.495%) and lower surface hydrophobicity. Moreover, the FP–CA complexes had better antioxidant activities than that of FP. Therefore, this study provides more insights for the potential application of FP–CA covalent complexes in functional food processing.

## 1. Introduction

Plant proteins have been widely applied in food processing and medical areas due to their health benefits, low cost and easy availability [1]. Flaxseed protein (FP) is a kind of plant protein extracted from defatted flaxseed cakes, which is rich in essential amino acids, including valine, leucine and isoleucine, and has a lower content of aromatic amino acids such as phenylalanine and tyrosine [2]. As the amino acid composition of FP contributes to a high ratio of branched-chain amino acids/aromatic amino acids, FP could resist cancer, burns, liver failure and other diseases [3]. However, FP exhibits a limited antioxidant activity and water-holding capacity (WHC), which restricts its broad application in the food industry. Therefore, it is urgent to select natural phenolic acids to construct FP–phenolic acid complexes to improve the antioxidant activity and functional properties of FP in food development.

Phenolic acids have various biological activities such as antioxidant, anti-inflammatory, antiviral, anticancer and antitumor activities [4]. Phenolic acids have attracted attention in modifying the structure of proteins to improve the protein function via binding with protein [5]. Protein–phenolic acid complexes can be formed via non-covalent interactions and covalent interactions to improve the functional properties of proteins [6,7]. These types of non-covalent interactions include hydrogen bonding, hydrophobic interactions, electrostatic interactions and van der Waals interactions [8]. Protein–phenolic acid covalent complexes are more stable in food applications than those formed by non-covalent interactions. The technologies for fabricating protein–phenolic acid covalent complexes mainly include free radical, enzymatic and alkaline methods [9]. The alkaline method as a nonenzymatic method is the most widely applied in fabricating protein–phenolic acid covalent complexes due to its low cost and convenience [10]. Polyphenols can be oxidized into electrophilic quinone compounds in an alkaline solution, and quinones further react with amino and thiol groups in proteins to form stable C–N or C–S covalent bonds [11]. Han et al. found that the emulsifying activity and stability of soy protein isolate were improved via covalent binding with Epigallocatechingallate (EGCG) [12]. The covalent interaction of the soy protein isolate and chlorogenic acid (CA) also enhanced the emulsifying activity and antioxidant activity of the soy protein isolate [13]. CA as one of common phenolic acids, is widely found in coffee, tea, vegetables and other medical plants [14]. CA is formed via the esterification of caffeic acid and quinic acid, which could display good antioxidant activity and medical value. Previous studies proved that CA had the potential to change the structure and improve the function of sunflower protein, whey protein and casein [15,16]. However, few studies on the structure characterization and functional properties of FP–CA complexes are available.

In this study, FP–CA covalent complexes were prepared using the alkaline method. The loading capacity and encapsulation efficiency (EE) of CA were first analyzed. The particle size, ζ–potential, turbidity, microstructure, Fourier transform infrared spectroscopy (FTIR) and thermal stability of FP–CA complexes were characterized. In addition, the effects of CA on the functional properties of the FP–CA complexes, including thermal stability, WHC, oil-holding capacity (OHC), emulsifying activity index (EAI) and antioxidant activity, were also evaluated.

## 2. Materials and Methods

### 2.1. Materials

The FP (purity 70%) was purchased from Shanxi Zhenghui Biotechnology Co., Ltd. (Xian, China), and FP without the addition of CA was chosen as the control group. The CA was purchased from Chengdu Biopurify Phytochemicals Ltd. (Chengdu, China). The 8–anilino–1–naphthalenesulfonic acid ammonium salt (ANS), 2,2–diphenyl–1–picrylhydrazyl (DPPH), ABTS and 2,4,6–tripyridyl–s–triazine (TPTZ) were purchased from Shanghai Yuanye Biotechnology Co., Ltd. (Shanghai, China). The CA was of HPLC grade, while the other reagents were of analytical grade.

### 2.2. Preparation of FP–CA Covalent Complexes

According to the method reported by Wu et al. with slight modifications [17], FP (100 mg) was dispersed in 20 mL distilled water to obtain a 5 mg/mL FP solution. The pH of the FP was adjusted to 11 using NaOH solution, and heated at 80 °C for 10 min to completely dissolve the FP. Different concentrations of CA solution were added to the FP solution with CA/FP mass ratios of 0.35%, 0.70%, 1.05% and 1.40% when the concentration of FP was kept at 5 mg/mL. The mixture was stirred at 400 rpm/min for 30 min using a magnetic stirrer. After the mixture was centrifuged at 10,000 rpm for 10 min, the precipitate was freeze-dried under a vacuum to obtain different FP–CA covalent complexes (FP–0.35%CA, FP–0.70%CA, FP–1.05%CA and FP–1.40%CA complex). FP alone was selected as the control group.

### 2.3. EE and Loading Capacity of CA in FP–CA Complexes

The FP–CA complexes were prepared according to the method described in Section 2.2. After the FP–CA complex solution was centrifuged at 10,000 rpm for 8 min, the supernatant was retained and diluted to an appropriate level. The absorbance of the supernatant was measured at 325 nm using a UV–Vis spectrophotometer (UV–2600, Shimadzu Instruments Co., Ltd., Tokyo, Japan). The standard curve of CA was plotted with the concentration of CA standard solution as x and the absorbance of CA as y. The EE and loading capacity of CA were calculated according to Formulas (1) and (2) [18].

(1) $$\mathrm{EE}\;\left(\%\right)=\left(1-\frac{m}{m_{0}}\right)\times 100\%$$

where *m* is the CA mass (mg) in the supernatant of the centrifuged FP–CA complex solution and *m*_0_ is the initial added CA mass (mg).

(2) $$Loading\;capacity\;\left(\%\right)=\frac{m_{0}-m}{m_{2}}\times 100\%$$

where *m* is the CA mass (mg) in the supernatant of the centrifuged FP–CA complex solution, *m*_0_ is the initial added CA content (mg) and *m*_2_ is the mass (mg) of the FP–CA complexes.

### 2.4. Particle Size, Ζ-Potential and Polydispersity Index (PDI)

The particle size, ζ–potential and PDI of the FP–CA complexes (FP–0.35% CA, FP–0.70% CA, FP–1.05% CA and FP–1.40% CA complex) were measured using a laser particle size analyzer (BeNano–90–Zeta, Baxter Instrument Co., Ltd., Dandong, China). The concentration of all the complexes was kept at 1 mg/mL. The refractive indexes for the FP–CA complexes and the dispersion medium were 1.46 and 1.33, respectively.

### 2.5. Turbidity

The turbidity of the 2 mg/mL FP solution and different FP–CA complex solutions (FP–0.35% CA, FP–0.70% CA, FP–1.05% CA and FP–1.40% CA complex) were measured at 600 nm using a UV–Vis spectrophotometer (UV–2600, Shimadzu Corporation, Tokyo, Japan) at 25 °C.

### 2.6. UV–Vis Spectra

UV–Vis spectroscopy (UV–2600, Shimadzu Corporation, Tokyo, Japan) was used to analyze the interaction between FP and CA. The FP protein solution (0.2 mg/mL), CA solution (50 μM) and their mixture were scanned at the wavelength range of 200–500 nm.

### 2.7. Fluorescence Spectra

The fluorescence intensities of CA, FP and the FP–CA complexes were scanned at an excitation wavelength of 280 nm and an emission wavelength range of 300–500 nm by a Cary Eclipse fluorescence spectrophotometer (Agilent Technologies, Santa Clara, CA, USA). The slit width for excitation and emission was both set at 5.0 nm, and the scanning speed was kept at 240 nm/min with the voltage of 400 V. The concentrations of the FP and FP–CA complex (FP–0.35% CA, FP–0.70% CA, FP–1.05% CA and FP–1.40% CA complex) solutions were both kept at 1 mg/mL. The fluorescence intensity of CA at 100 μM was also measured.

### 2.8. FTIR Spectra

The FTIR spectra of FP and the FP–CA complexes were determined by an FTIR spectrophotometer (VERTEX70, Bruker Optics Inc., Ettlingen, German). KBr was mixed with CA, FP and the FP–0.35%CA complex at a ratio of 100:1 (*w*/*w*), respectively. The wavelength range was set as 4000–400 cm^−1^ with the resolution of 4 cm^−1^ and the number of scans set as 32 [19].

### 2.9. Differential Scanning Calorimetry (DSC)

DSC (METTLER–TOLEDO, Schwerzenbach, Switzerland) was adopted to determine the thermal stability of FP and the FP–0.35%CA complexes. The temperature was increased from 25 °C to 250 °C with the heating rate of 10 °C/min, and the nitrogen flow rate was 100 mL/min [20]. The FP–0.35%CA complex, FP and CA (5.0 mg) were sealed in aluminum crucibles, with an empty aluminum crucible as the control.

### 2.10. Scanning Electron Microscopy (SEM)

SEM (TM3030Plus, Co., Tokyo, Japan) was used to observe the microstructure of FP–CA complexes and FP. The freeze-dried FP and FP–0.35% CA complex were fixed on the sample stage with conductive double-sided tape. After blowing the excess sample, the samples were gold sputtered under vacuum and then observed under SEM at an accelerating voltage of 15 kV.

### 2.11. WHC Analysis

The WHC of FP and the FP–CA complexes was measured according to the method that Jing et al. reported with some modification [21]. FP and the FP–CA complexes (40 mg) were added to a 2 mL centrifuge tube, followed by the addition of 2 mL distilled water. The mixture was shaken for 10 min in a vortex and then centrifuged at 10,000 r/min for 15 min. The centrifugated supernatant was weighed, and the WHC of FP and the FP–CA complexes was calculated using the following formula:

(3) $$WHC\;\left(\%\right)=\frac{{m_{1}-m}_{2}}{m}\times 100\%$$

*m*_1_: the mass of the added distilled water, g;
*m*_2_: the mass of the centrifugated supernatant, g;
*m*: the mass of the FP or FP–CA complexes, g.

### 2.12. OHC Analysis

The OHC of FP and the FP–CA complexes was measured according to the method reported by Shen et al. with some modification [22]. The FP–CA complexes (40 mg) were added to a 2 mL centrifuge tube, followed by the addition of 2 mL peanut oil. The mixture was vigorously shaken for 10 min and then centrifuged at 10,000 r/min for 15 min. The upper oil was weighed, and the OHC of FP and the FP–CA complexes was calculated by the following formula:

(4) $$OHC\;\left(\%\right)=\frac{{m_{1}-m}_{2}}{m}\times 100\%$$

*m*_1_: the mass of the added peanut oil, g;
*m*_2_: the mass of the centrifugated upper oil, g;
*m*: the mass of the FP or FP–CA complexes, g.

### 2.13. Emulsifying Activity Index (EAI) Analysis

The EAI of FP and the FP–CA complexes was determined based on the method described by Han et al. with some modification [12]. FP and the FP–CA complexes were firstly mixed with soybean oil at the ratio of 1:3. The mixture was further homogenized for 10 min and diluted 100 times with 0.1% (*w*/*w*) sodium dodecyl sulfate solution. The absorbance of the diluted solution at 500 nm was measured. The EAI of FP and all the complexes was calculated using the following formula:

(5) $$\mathrm{EAI}\;({\mathrm{cm}}^{2}\cdot g^{-1})=\frac{2\times 2.303\;\times \;A_{0}\times D\times 10^{3}}{\varphi \times L\times C\times 10^{4}}$$

where D is the dilution factor, C is the protein concentration (mg/mL), L is the path length of the colorimeter (1 cm), *φ* is the proportion of the oil phase (0.25) and A_0_ is the absorbance of the sample.

### 2.14. Surface Hydrophobicity Analysis

According to the method that Han et al. reported [23], the FP and FP–CA complex solutions (3 mL) at 1 mg/mL were mixed with 30 μL of 8 mM ANS solution, respectively. The mixture was reacted in the dark for 5 min. Finally, the fluorescence intensity of the sample was measured by using a Cary Eclipse fluorescence spectrophotometer (Agilent Technologies, Santa Clara, CA, USA) with an excitation wavelength of 390 nm and an emission wavelength of 470 nm.

### 2.15. Antioxidant Activity Analysis

#### 2.15.1. DPPH Radical Scavenging Capacity Assay

Based on the method reported by Parolia et al. with some modification [24], the FP and FP–CA complex solutions (1 mg/mL) and 0.1 mM DPPH solution were prepared. The FP and FP–CA complex solutions (100 μL) at 1 mg/mL were mixed with 900 μL of DPPH solution (0.1 mM), respectively. The mixture was reacted in the dark for 10 min. The absorbance at 517 nm was measured. The DPPH radical scavenging capacity of samples was calculated as follows:

(6) $$DPPH\;scavenging\;rate\;\left(\%\right)=(1-\frac{A_{s}}{A_{0}})\times 100\%$$

where A_s_ is the sample absorbance and A_0_ is the control absorbance.

#### 2.15.2. Ferric Reducing Antioxidant Power (FRAP) Assay

Referring to the method reported by Qie et al. [25], the FP and FP–CA complex solutions (100 μL) at 1 mg/mL were mixed with 900 μL of FRAP, respectively, and the mixture was reacted in the dark for 20 min. Vitamin C (Vc) was selected to make the standard curve. The absorbance of the mixture was measured at 593 nm. The FRAP values of samples were expressed as Vc μg/mL.

#### 2.15.3. ABTS Radical Scavenging Capacity Assay

According to the method reported by Jiang et al. [26], the FP and FP–CA complex solutions at 1 mg/mL were mixed with 900 μL of ABTS reagent, respectively, and the mixture was reacted in the dark for 20 min. The absorbance of the mixture was measured at 734 nm. The ABTS radical scavenging capacity was calculated using the formula:

(7) $$ABTS\;scavenging\;rate\;\left(\%\right)=(1-\frac{A_{s}}{A_{0}})\times 100\%$$

where A_s_ is the sample absorbance and A_0_ is the control absorbance.

### 2.16. Data Analysis

All the experiments were performed in triplicate, and the results were expressed as mean ± standard deviation. Statistical analysis was performed by using SPSS 16.0. Significant differences were analyzed using Tukey’s test. *p* < 0.05 was considered to be statistically significant.

## 3. Results and Discussion

### 3.1. EE and Loading Capacity of the FP–CA Complexes

**Figure 1:**
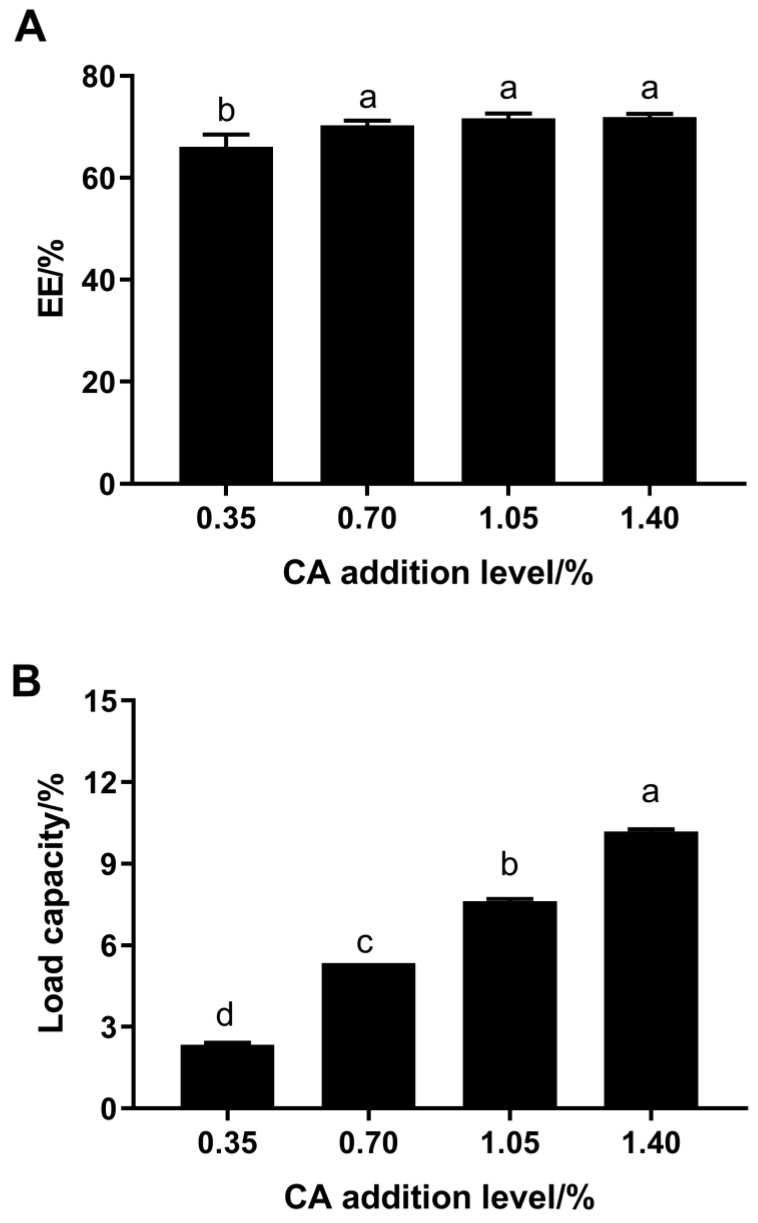
EE (**A**) and loading capacity (**B**) of the CA in the FP–CA complexes with 0.35%. Different letters indicate significant differences (*p* < 0.05).

The EE and loading capacity of the FP–CA complexes are shown in Figure 1. The EE of the FP–CA complexes with 0.70–1.40% CA ranged from 66.11% to 72.20%, which was significantly higher than that of the complex with 0.35% CA. However, there was no significant difference on the EE of the complexes with 0.70–1.40% CA. The loading capacity of the FP increased with the increasing addition ratio of CA in a dose-dependent relationship, which ranged from 2.34% to 10.18% (Figure 1B). Similar results were also reported that the amount of catechin loaded by the pea protein isolate increased with the addition of catechin [27]. These results indicated that the FP had a high CA-loaded capacity, and excessive CA could not be encapsulated by FP.

### 3.2. Particle Size, PDI and Ζ-Potential Analysis

**Figure 2:**
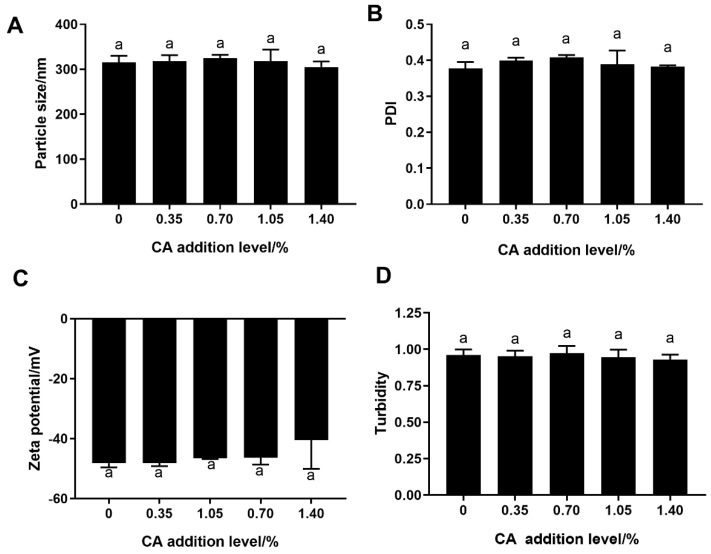
The particle size (**A**), PDI (**B**), ζ–potential (**C**) and turbidity (**D**) of FP and the FP–CA complexes with different CA levels (0.35%, 0.70%, 1.05% and 1.40%). Different letters indicate significant differences (*p* < 0.05).

Figure 2 shows the particle size, PDI and ζ–potential of FP and the FP–CA complexes. The particle size of FP and the FP–CA complexes with different CA addition levels (0, 0.35%, 0.70%, 1.05% and 1.40%) were 315.69, 318.29, 324.51, 305.47 and 305.25 nm (Figure 2A), respectively. The PDI of FP and the FP–CA complexes with different CA addition levels (0, 0.35%, 0.70%, 1.05% and 1.40%) were 0.38, 0.40, 0.41, 0.39 and 0.38, respectively (Figure 2B). However, there were no significant differences in the particle size and PDI of FP and the FP–CA complexes with different CA/FP ratios. The above results indicated that the addition of 0.35–1.40% CA did not affect the size and uniformity of particle distribution of the FP, and no larger FP–CA complex aggregates were formed. The soy protein isolate binding with catechin in lower concentrations also formed a similar particle size [28].

The potential of FP and the FP–CA complexes is shown in Figure 2C. The potential values of FP and the FP–CA complexes with different CA/FP ratios (0.35%, 0.70%, 1.05% and 1.40%) are −48.16, −48.11, −46.40, −46.55 and −40.43 mV, respectively. There were no significant differences in ζ-potential between FP and the FP–CA complexes. The FP and FP–CA complexes had negative surface potentials, and CA addition did not affect the electrostatic repulsion between particles. Moreover, the absolute values for all the samples were larger than 40 mV, indicating that both FP and the FP–CA complexes had a high stability [29], which was related to the strong electrostatic repulsion between adjacent particles. Therefore, the covalent interaction between CA and FP could not change the particle size, PDI and ζ−potential of the FP–CA complexes.

### 3.3. Turbidity Analysis

Figure 2D shows the turbidity of FP and the FP–CA complexes. The turbidity of FP and the FP–CA complexes with 0.35–1.40% CA ranged from 0.93 to 0.96. There was no significant difference in turbidity between FP and the FP–CA complexes with different CA levels, which was consistent with the results of particle size and PDI. These results further indicated that different addition levels of CA binding with FP did not affect the collision and aggregation between the FP–CA complex molecules. However, the result was not contrary to the previous study that the turbidity of soybean protein isolate was increased due to its covalent binding with catechin [12], which might be caused by the different interaction forces between the FP–CA complexes and the soybean protein isolate–catechin complexes.

### 3.4. UV–Vis and Fluorescence Spectrum Analysis

**Figure 3:**
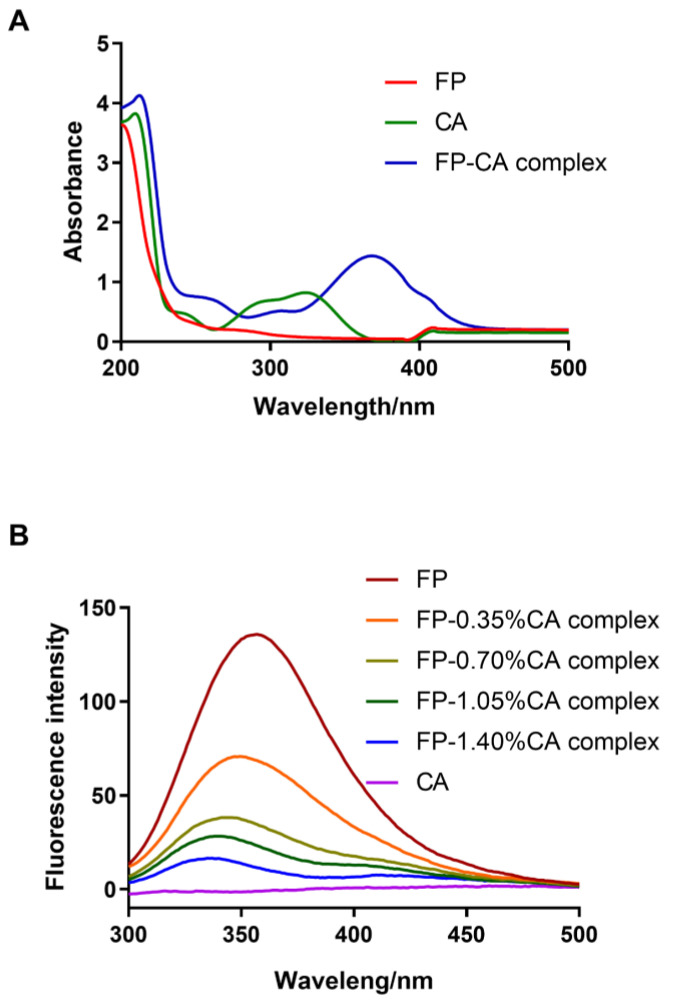
The UV–Vis (**A**) and fluorescence spectra (**B**) of CA, FP and the FP–CA complexes.

UV–Vis and fluorescence spectra can be used to reveal the interaction between FP and CA. The maximum absorption peak of the FP is around 280 nm in Figure 3A, as the FP contained aromatic amino acids, including tyrosine, tryptophan and phenylalanine [30]. The maximum absorption peak of CA was around 325 nm. The FP–CA complex displayed the maximum absorption wavelength around 375 nm, due to the covalent binding of CA and FP. These results revealed that the interaction between FP and CA led to a red shift of the maximum absorption peak of CA. The addition of astaxanthin also caused the shift of the maximum absorbance peak of β-lactoglobulin [31]. Figure 3B shows the fluorescence spectra of FP and the FP–CA complexes with different CA levels. FP had the highest fluorescence intensity at the excitation wavelength of 280 nm, and the fluorescence intensity of the FP–CA complexes gradually decreased as CA addition level increased. The fluorescence of the FP was quenched by CA due to the fluorescence chromophore of FP reduced by the interaction between FP and CA. Hao et al. [23] also reported that EGCG, CA and resveratrol could quench the fluorescence intensity of the pea protein isolate via the non-covalent interaction between polyphenols and the pea protein isolate. Moreover, the maximum emission wavelength of FP at the excitation wavelength of 280 nm exhibited a blue shift from 351 nm to 335 nm. Both the red shift of the maximum UV absorption peak of CA and the fluorescence of FP quenched by CA verified the interaction between FP and CA.

### 3.5. FTIR Analysis

**Figure 4:**
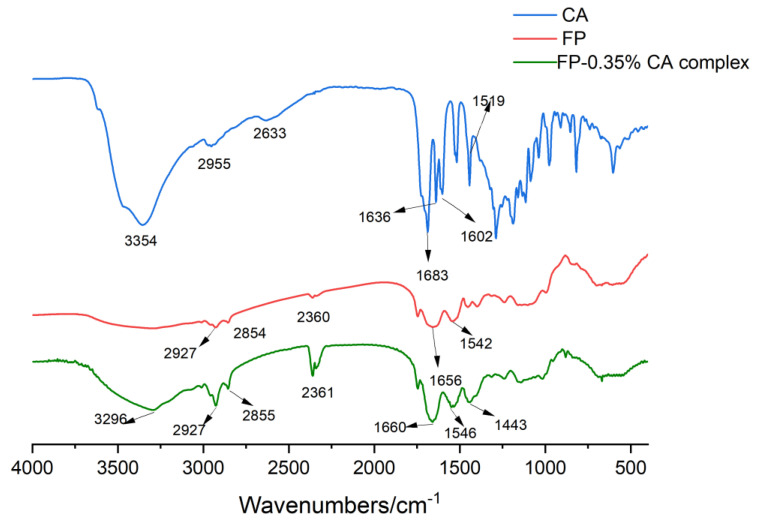
FTIR of CA, FP and the FP−0.35%CA complex.

FTIR can provide information about the chemical structural changes of proteins by detecting the position of characteristic peaks [32]. The infrared spectra of CA, FP and the FP–0.35%CA complex are shown in Figure 4. The infrared spectrum of FP exhibited a peak at 1656 cm^−1^ (amide I band, the stretching vibration of C=O), 1542 cm^−1^ (amide II band, N–H bending and C–N stretching) and characteristic peaks at 2854 cm^−1^ and 2927 cm^−1^ (C–H stretching) [33]. CA showed peaks at 1683 cm^−1^, 1636 cm^−1^, and 1602 cm^−1^ (C=O stretching vibration), 1519 cm^−1^ (N–H bending and C–N stretching) and 2955 cm^−1^ (C–H stretching). Moreover, the peak near 3354 cm^−1^ of CA was attributed to the O–H stretching of hydroxyl groups. The FP–0.35%CA complex exhibited peaks at 1660 cm^−1^ and 1546 cm^−1^, which resulted from the red shift of amide I band and amide II band in FP spectra. The characteristic peak at 3296 cm^−1^ of the FP–0.35%CA complex was attributed to the blue shift of the characteristic peak of CA (3354 cm^−1^). A similar phenomenon was also found in curcumin in cellulose nanocrystals nanoparticles [34]. The peak of CA at 2955 cm^−1^ disappeared in the spectra of the FP–0.35%CA complex. These results indicated that CA interacted with FP and caused the second structure of FP, which could affect the WHC, OHC and other functional activities of FP.

### 3.6. Thermal Stability

**Figure 5:**
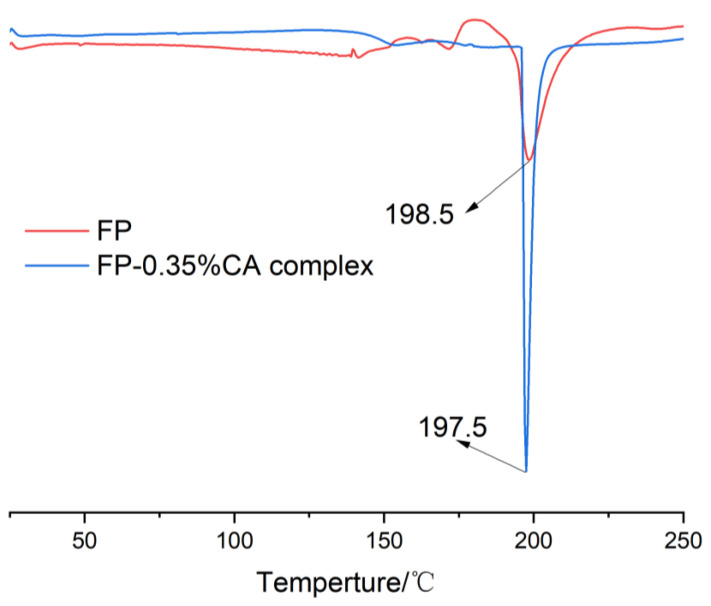
Thermal stability of FP and the FP–0.35%CA complex.

DSC was adopted to evaluate the thermal stability of FP and the FP–0.35%CA complex. Figure 5 shows that the decomposition temperature of FP at 198 °C was higher than that of the FP–0.35%CA complex (197 °C), which implied that the interaction between FP and CA could change the FP structure and the stability of the FP–0.35%CA complex declined. Han et al. found that the thermal stability of pea protein isolate–EGCG complexes was lower than that of the pea protein isolate alone [35]. Liu et al. found that the denaturing temperature of the zein was higher than that of the zein–EGCG complexes [36]. A previous study reported that the thermal stability of the FP–phenolic acid adducts decreased after removing the phenolic acids [37]. Therefore, the covalent combination of FP and CA would reduce the stability of FP in food applications.

### 3.7. SEM Results

**Figure 6:**
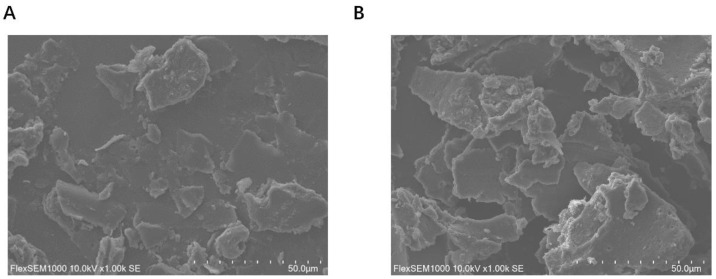
SEM images of FP (**A**) and the FP–0.35%CA complex (**B**).

SEM can provide the visual morphology and surface features of protein. In Figure 6, both FP and the FP–0.35%CA complex exhibit an irregular sheet-like structure, as they underwent the conformational change and rearrangements of their structure during vacuum freeze-drying. However, the FP–0.35%CA complex had more wrinkles on the surface compared to the FP, which might be attributed to the interaction forces between hydroxy groups and amino acid residues in FP. Similar wrinkles were also found on the surface of Moringa oleifera leaf polyphenol-rich extract microencapsulated with maltodextrin [38]. The microstructure of the pea protein was also changed due to its binding with curcumin [20]. These results proved that the microstructure of FP was slightly changed due to the covalent binding between CA and FP.

### 3.8. WHC

**Figure 7:**
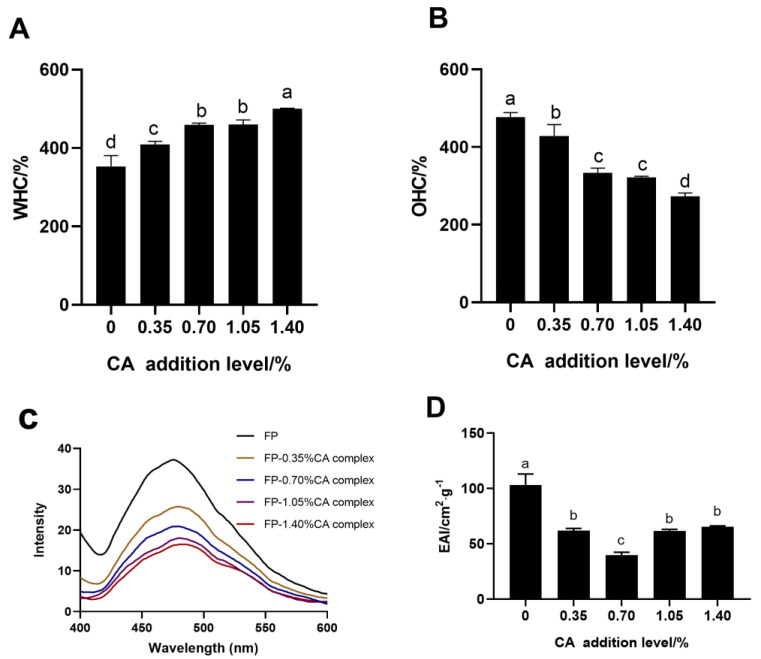
The WHC (**A**), OHC (**B**), surface hydrophobicity (**C**) and EAI (**D**) of FP and FP–CA complexes with 0.35%, 0.70%, 1.05% and 1.40% levels of CA. Different letters indicate significant differences between samples (*p* ≤ 0.05).

WHC represents the capacity of FP to retain water. As shown in Figure 7A, the WHC values of the FP–CA complexes are all significantly higher than that of FP. The WHC of the FP–CA complexes with different CA levels of 0.35%, 0.70%, 1.05% and 1.40% was 409.05%, 459.29%, 460.16% and 500.81%, respectively, which were all higher than that of FP (353.03%). These results indicated that the hydrophilic hydroxyl groups on CA contributed to the ability of FP binding with water. Moreover, the binding of FP and CA might induce the exposure of hydrophilic groups to the environment [39]. Previous studies also showed that the WHC of the whole bean protein isolate with phenolics was higher than that of whole bean protein isolate free of soluble phenolics [40]. Therefore, CA could improve the WHC of the FP–CA complexes and enhance the solubility of the FP-enriched food.

### 3.9. OHC

As shown in Figure 7B, the OHC of the FP–CA complexes is significantly reduced by the addition of CA in a dose–manner effect. The OHC values of the FP–CA complexes with a CA addition level of 0.35–1.40% ranged from 428.525% to 273.495%, which were all lower than that of FP alone. These results were consistent with the findings of the WHC of the FP–CA complexes with different levels of CA. Previous studied suggested that the surface hydrophobicity of the pea protein–EGCG complexes decreased due to the binding with EGCG, which could further weaken the OHC of the pea protein [23]. Due to the interaction between CA and the FP, the structure of the FP–CA complexes was changed and hydrophobic groups on FP were masked, which might lead to the decreased OHC of FP.

### 3.10. Surface Hydrophobicity

Surface hydrophobicity is an important indicator of the function properties of protein. The fluorescence intensity of the FP–ANS and complex–ANS represents their surface hydrophobicity. As shown in Figure 7C, the fluorescence intensity of FP–ANS is the highest, suggesting that the hydrophobicity of FP alone is higher than that of all the FP–CA complexes. Previous study also showed that hydroxytyrosol could lower the surface hydrophobicity of the FP–hydroxytyrosol complex [41]. Moreover, gallic acid also decreased the surface hydrophobicity of the beta lactoglobulin [42]. The surface hydrophobicity of the FP–CA complexes decreased with the increased levels of CA, which was attributed to that CA might interact with the hydrophobic amino acid residues on the FP surface and reduce the exposure of hydrophobic amino acids. Moreover, the hydrophilic hydroxyl groups on CA were introduced into FP, which further weakened the surface hydrophobicity of the FP–CA complexes.

### 3.11. Emulsifying Properties

Proteins are commonly selected as emulsifiers to reduce surface tension of liquid interfaces and promote stability of emulsion droplets. The EAI values of FP and the FP–CA complexes are shown in Figure 7D. The EAI values of the FP–CA complexes were all lower than that of FP alone. There were no significance differences in the EAI of the FP–CA complexes with a CA level of 0.35%, 1.05% and 1.40%. FP with 0.70% CA exhibited the lowest EAI (39.70 cm^2^·g^−1^), which suggested that CA decreased 61.52% of the EAI of FP. The lentil protein conjugating with the onion skin phenolics lowered the emulsifying properties of the lentil protein–onion skin phenolic complex [43]. A similar phenomenon was also reported on the EAI of the FP–hydroxytyrosol complex [41]. CA might alter the structure of FP and lower its capacity to absorb to the oil–water interface. Therefore, the FP–CA complexes were not suitable as emulsifiers for fabricating emulsion.

### 3.12. Antioxidant Activity

Constructing protein–polyphenol complexes is an effective strategy to enhance the antioxidant activity of protein [5]. The antioxidant activity of FP and the FP–CA complexes is shown in Table 1. The ABTS radical scavenging activity of the FP–CA complexes significantly increased with the increased levels of CA (Table 1). The ABTS radical scavenging activity of the FP–CA complexes with 0.35%, 0.70%, 1.05% and 1.40% CA was 42.77%, 49.66%, 54.09% and 52.61%, respectively. However, there was no significant difference in the ABTS radical scavenging activity of the FP with 1.05% CA and 1.40% CA. The CA levels ranging from 0.35% to 1.40% increased the ABTS radical scavenging activity of FP by 18.83–50.30%.

The FRAP of the FP–CA complexes significantly increased with the increased levels of CA in a significant dose–response relationship (Table 1). The FRAP of the FP–CA complexes with 0.35–1.40% CA was 17.21–56.94 μg V_C_/mL, which was 2.33–7.22 times as much as that of FP. Previous studies also proved that the in vitro antioxidant activity of PP was enhanced after conjugation with phenolic acid [5]. The antioxidant activity of the soy protein–EGCG conjugation was also higher than that of soy protein [44]. The FRAP of the lentil protein–phenolic acid conjugates was also higher than that of the lentil protein and depended on the type of phenolic acids [24].

Table 1 shows that the DPPH scavenging activity of FP had no discrepancy with that of the FP–CA complexes with the CA level of 0.35–1.05%. However, the DPPH scavenging activity of the FP–CA complex with 1.40% CA increased by 309.51%, compared with FP alone. This result was in agreement with the fact that the DPPH radical scavenging activity of the LF–70 μM CA complex increased by 434.65% compared with the LF [45]. The increase in the FRAP, DPPH and ABTS radical scavenging activity of the FP–CA complexes was attributed to the hydroxyl groups introduced into FP [46]. These results indicated that CA could improve the antioxidant properties of FP.

## 4. Conclusions

This study revealed that the structure and functional properties of the FP–CA complexes were affected by the covalent interaction between FP and CA. FP could reach the highest EE (72.20%) and loading capacity (10.19%) of CA, suggesting that FP could be selected as a good carrier to load CA. CA binding with FP did not change the particle size, turbidity, ζ–potential and PDI of FP–CA complexes. The interaction between FP and CA made the FP–CA complexes show more wrinkles on the surface and have a lower stability. It is worth noting that the stability of CA in food enriched with the FP–CA complex would be reduced during food processing. The WHC of the FP–CA complexes was higher than that of FP alone while their OHC and surface hydrophobicity became lower. Moreover, the DPPH scavenging activity, ABTS radical scavenging activity and FRAP of the FP–CA complexes were enhanced by the high addition level of CA. These results provided more evidence on the application of the FP–CA complexes in functional food to broad the utilization of polyphenols and plant proteins.

## Figures and Tables

**Table 1:** The ABTS scavenging activity, FRAP and DPPH scavenging activity of FP and the FP–CA complexes with 0.35%, 0.70%, 1.05% and 1.40% levels of CA.

CA Addition Level/%	DPPH Radical Scavenging Activity/%	ABTS Radical Scavenging Activity/%	FRAP
0.00	2.53 ± 0.25 ^b^	35.99 ± 0.87 ^d^	7.76 ± 0.10 ^e^
0.35	2.80 ± 0.09 ^b^	42.77 ± 0.87 ^c^	17.21 ± 0.15 ^d^
0.70	2.88 ± 1.07 ^b^	49.66 ± 1.48 ^b^	36.19 ± 0.33 ^c^
1.05	2.69 ± 0.09 ^b^	54.09 ± 1.46 ^a^	47.18 ± 0.30 ^b^
1.40	7.82 ± 1.27 ^a^	52.61 ± 1.35 ^a^	56.94 ± 1.21 ^a^

Different letters indicate significant differences between samples (*p* ≤ 0.05).

## Data Availability

The datasets generated for this study are available on request to the corresponding author. The data are not publicly available due to the protection of this article copyright.
